# The Relationship Between Barriers to Medical Error Disclosure and Patient Safety Culture: A Cross‐Sectional Study

**DOI:** 10.1155/jonm/9980576

**Published:** 2026-04-07

**Authors:** Gui-Ru Chen, Xin Luo, Rong-Rong Huang, Heng-Yu Xiong, Xiao-Min Ding, Shi-Hua Pan

**Affiliations:** ^1^ Department of Infectious Diseases, The People’s Hospital of Aba Tibetan and Qiang Autonomous Prefecture, Maerkang, Sichuan, 624000, China, scu.edu.cn; ^2^ Operating Room, The People’s Hospital of Aba Tibetan and Qiang Autonomous Prefecture, Maerkang, Sichuan, 624000, China, scu.edu.cn; ^3^ Department of Burn and Plastic Surgery, The Affiliated Hospital of Guizhou Medical University, Guiyang, 550001, China, gmcah.cn; ^4^ Department of Nursing Department, Sichuan Provincial People’s Hospital, University of Electronic Science and Technology of China, Chengdu, 610072, China, uestc.edu.cn; ^5^ Department of Neuropsychology, Zhongnan Hospital of Wuhan University, Wuhan, 430071, China, znhospital.cn; ^6^ Department of General Medicine, Zhongnan Hospital of Wuhan University, Wuhan, 430071, China, znhospital.cn

## Abstract

**Background:**

Changes in the patient safety culture in hospitals can increase error disclosure, but the relationship between these factors remains unclear.

**Objectives:**

To explore nurses’ perceptions of barriers to the disclosure of medical errors in the context of a patient safety culture and to identify relevant influencing factors.

**Design:**

Cross‐sectional study.

**Settings:**

Two tertiary and two secondary comprehensive hospitals in southwestern China.

**Participants:**

Chinese registered nurses who were employed in secondary or tertiary comprehensive hospitals.

**Methods:**

In May 2023, electronic questionnaires were distributed to all nurses in the selected hospitals. The survey included nurses’ characteristics, a hospital survey on patient safety culture, and the Chinese version of the barriers to error disclosure assessment (C‐BEDA) tool. The data thus collected were subjected to descriptive and multiple linear regression analyses.

**Results:**

The effective recovery rate was 96.23% (792/823). Nurses reported that financial concerns represented the greatest barrier to medical error disclosure, followed by psychological barriers and knowledge barriers. A multiple linear regression analysis revealed that working departments, communication openness, feedback and communication about errors, nonpunitive responses to errors, teamwork across units, and hand‐offs and transitions collectively accounted for 27.2% of the variation in nurses’ confidence and knowledge barriers. Age, title, managerial position, tenure, marital status, and continuous improvement in organizational learning explained 28.7% of the variation in the institutional barriers faced by nurses. Gender, title, tenure, supervisor/manager expectations, actions taken to promote patient safety, and openness to communication explained 10.2% of the variation in the psychological barriers faced by nurses.

**Conclusions:**

To encourage nurses to disclose medical errors effectively, hospitals must first address nurses’ concerns regarding economic issues and provide them with comprehensive training in error disclosure alongside psychological support. Hospitals should prioritize the establishment of a patient safety culture at the unit level, particularly with respect to organizational learning, continuous improvement, and openness to communication.

## 1. Introduction

Medical errors are errors or mistakes that are committed by health professionals and result in harm to patients. Medical errors represent honest mistakes or accidents, such as diagnostic errors or errors in the administration of drugs and other medications. The occurrence of medical errors as a result of unsafe care is likely one of the 10 leading causes of death and disability worldwide and has come to represent a serious global public health concern [1].

Following a medical error, the protection of the rights of patients and their families, as the primary victims, should be prioritized [2]. Health professionals who are involved in such errors, who thus experience adverse effects and are viewed as second victims, have received increasing attention in this context [2, 3]. Nurses are the most common second victims of such events and suffer serious psychological, physical, and occupational injuries in this context [2, 4, 5]. The healthcare institution in which the error occurred may face reputational and financial repercussions, thus identifying it as the third victim [6, 7]. Future patients seeking medical care represent potential fourth victims [8]. Stakeholders who are affected by medical errors are clearly multifaceted; thus, a balanced approach to the task of safeguarding human rights and safety from various perspectives is required.

Medical error disclosure refers to situations in which healthcare institutions proactively inform patients of mistakes that have occurred. This behaviour includes discussing the events that have transpired; elucidating the relationship between the error and its consequences; explaining the implications for the patient; and negotiating remedial measures, compensation, and an apology. Disclosure itself thus represents an assertion of priority with regard to the rights of the first victim. When hospitals disclose and communicate mistakes openly, treat patients in a timely manner, and improve their relationships with patients, disclosure becomes a way in which they can heal the harm caused to second victims [9]. A prerequisite for disclosing errors is to admit them, which can greatly promote the transformation and improvement of hospital organizations, thereby transforming the disclosed medical responsibility into practical action.

The disclosure of medical errors has been viewed as a fair and transparent approach that not only actively protects patients’ rights but also plays a positive role in efforts to alleviate the harm caused to second victims and to promote continuous improvement for third victims. This approach can effectively ensure patient safety and facilitate the transformation of the doctor‒patient relationship in the new era from a focus on subjective trust‐disappointment to an emphasis rule–based trust compensation. However, error disclosure is not universal at the global level.

Surveys have revealed that approximately 49%–85% of medical errors are not disclosed by hospitals to victims [10–13]. Even in the virtual world, such as in television dramas, nearly half of such mistakes are not disclosed to patients [14]. Error disclosure faces numerous barriers, including legal constraints, policy limitations, sociocultural factors, public perceptions, disclosure capabilities on the part of hospitals, and preparedness for disclosure, which collectively contribute to the limited prevalence of error disclosure [15–19]. In 2021, a tool for measuring barriers to the disclosure of medical errors in terms of psychological barriers, institutional barriers, and economic barriers was developed [20]. This tool has had a positive impact and can help hospitals implement disclosure more effectively in the context of clinical practice.

The disclosure of errors to patients is an organizational behaviour; thus, such disclosure is also influenced by the hospital’s patient safety culture. Inefficient reporting systems, the perceived adverse consequences of disclosure, the individual who made the mistake, the unequal status of that individual after the error is reported, and a lack of consensus concerning disclosure all seriously hinder hospitals’ efforts to disclose errors, which may be related to the hospital’s patient safety culture [21, 22]. Changes in the patient safety culture in hospitals can promote error disclosure [21], but the relationship between these two factors remains unclear. A hospital survey on patient safety culture (HSOPSC) was developed by the Agency for Healthcare Research and Quality (AHRQ), and this survey has primarily been used to evaluate the patient safety culture in hospitals. This tool can be used to produce a comprehensive evaluation of the patient safety culture that characterizes hospitals, to identify nurses’ perceptions of safety at different levels within both units and hospitals, and to explore the obstacles that can prevent the disclosure of medical errors at different levels.

Nurses represent the largest contingent among health professionals and bear primary responsibility for implementing medical directives. In the contexts of medication errors, blood transfusion mishaps, unplanned extubations, and similar incidents, nurses bear the critical responsibility of protecting patients’ lives and are obligated to disclose such errors to patients transparently. Additionally, given that nurses represent the second victims of these incidents, they also require support and attention from their superiors as well as researchers. This study, which is rooted in the broader context of hospital patient safety culture, examines nurses’ perceptions of the barriers to the disclosure of medical errors. By investigating the relationship between patient safety culture and these barriers to disclosure, this study provides theoretical foundations that hospitals can use to enhance nursing management continuously, address challenges pertaining to disclosure, and progressively implement clinical disclosure practices.

## 2. Materials and Methods

### 2.1. Study Design, Setting, and Participants

In May 2023, nurses from two tertiary grade‐A general hospitals and two secondary grade‐A hospitals in southwestern China were selected as research subjects using a convenience sampling approach. The inclusion criteria required participants to have obtained a nurse’s practice qualification certificate, be engaged in clinical nursing or management work, and be willing to participate in this study. Exclusion criteria: trainee nurses, visiting scholars, and those who had been absent from duty for a period exceeding 6 months during the survey from participating. The sample size was determined according to two methodologically grounded criteria: (1) adherence to the conventional 10:1 observation‐to‐variable ratio, yielding a minimum required sample of 270 participants for the 27 variables included in the analysis, and (2) application of cluster sampling methodology demands that all eligible nurses across the four participating hospitals be included as much as possible.

In the four included hospitals, no plan to disclose errors proactively had yet been implemented. When patients and their families demand the disclosure of errors, hospitals often implement passive disclosure plans. During the process of investigating the incident, hospitals communicate with patients and their families, obtain a full understanding of their demands, offer apologies, and negotiate compensation. Nurses often do not directly participate in such disclosures, but they may be held accountable for their faults, including through public criticism, administrative penalties, economic compensation, and participation in post‐event apologies. These hospitals are nonprofit public medical institutions. The purchase of medical liability insurance is not administratively mandatory. In this context, three hospitals had purchased medical liability insurance; thus, doctors and nurses in these hospitals were not required to pay for or purchase such insurance personally. The annual insurance premium expenditure is related to factors such as the number of doctors and nurses and the risk levels associated with different specialties. The annual expenditure accounts for approximately 500,000 to 1,000,000 RMB.

### 2.2. Variables

#### 2.2.1. Demographic and Work‐Related Characteristics

The following variables were collected: age, gender, education, professional title, position, marital status, form of labour contract, department, tenure, and hospital level. Reporting times for adverse events were collected via the HSOPSC, section G: In the past 12 months, how many event reports have you filled out and submitted? To ensure anonymity, sensitive participant information—including names, phone numbers, email addresses, and residential addresses—was not collected.

#### 2.2.2. The Chinese Version of the Barriers to Error Disclosure Assessment (C‐BEDA) Tool

The BEDA tool was developed by Welsh et al. [20]. This tool can be used to measure individuals’ perceptions of their ability to disclose, their impressions regarding institutional policies and climate, and specific barriers that can inhibit disclosure by health professionals. This tool consists of four dimensions, including barriers pertaining to confidence and knowledge, institutional barriers, psychological barriers, and financial concerns. Preliminary evidence has been reported in support of the tool’s validity and reliability regarding efforts to measure variables related to disclosure. The BEDA tool contains 31 items, most of which are scored on a five‐point Likert scale ranging from 1 (strongly disagree or not at all a barrier) to 5 (strongly agree or very much a barrier). The dimension score is equal to the sum of the scores assigned to each item divided by the total number of items. Higher scores indicate more barriers. The C‐BEDA tool was adapted by Huang et al. [23]; the item‐level content validity index for this tool ranged from 0.86 to 1.00, and the average scale‐level content validity index was 0.98. The Cronbach’s *α* coefficient (0.91) and test‐retest reliability (0.86) were acceptable.

#### 2.2.3. HSOPSC

HSOPSC has been reported to exhibit robust reliability and validity, and it has come into widespread use at the global level. In this study, the revised version of this survey developed by Xiao [24], which consists of 12 dimensions and 42 items, was used. Specifically, seven dimensions pertain to patient safety culture at the departmental level, and three dimensions assess safety culture at the hospital level, as indicated in Table 1. In addition, this measure features two outcome variables: overall perceptions of patient safety and the frequency of events reported. The items included in this measure are scored on a five‐point Likert scale. The dimension score is equal to the average score of the corresponding items. Higher scores indicate higher levels of awareness of patient safety culture among nurses. The Cronbach’s *α* coefficient (0.89) and split‐half reliability (0.78) in this study were acceptable.

**TABLE 1:** The score of the included variables.

Variables	Dimensions	Minimum	Maximum	Mean ± SD	Skewness	Kurtosis
Dimensions of the Barriers to Error Disclosure Assessment Tool	Confidence and knowledge barriers	1.00	4.60	2.62 ± 0.56	−0.09	0.83
	Institutional barriers	1.00	4.75	2.56 ± 0.59	−0.17	0.74
	Psychological barriers	1.00	5.00	3.13 ± 0.81	−0.62	0.16
	Financial concern barriers	1.00	5.00	3.28 ± 0.88	−0.39	−0.02

Dimensions of the unit level	U1 Supervisor/manager expectations & actions promoting patient safety	1.25	5.00	3.74 ± 0.57	−0.32	0.58
	U2 Organizational learning—continuous improvement	2.00	5.00	4.00 ± 0.45	−0.15	1.35
	U3 Teamwork within units	1.25	5.00	4.02 ± 0.53	−0.49	1.84
	U4 Communication openness	1.00	5.00	3.31 ± 0.69	0.03	0.28
	U5 Feedback & communication about error	1.00	5.00	3.86 ± 0.77	−0.48	0.17
	U6 Non‐punitive response to errors	1.00	5.00	3.22 ± 0.69	−0.52	0.88
	U7 Staffing	1.50	5.00	2.99 ± 0.61	0.14	0.03

Dimensions of the hospital level	H1 Management support for patient safety	1.67	5.00	3.90 ± 0.58	−0.22	0.19
	H2 Teamwork across units	1.75	5.00	3.78 ± 0.59	−0.03	0.03
	H3 Hand‐offs & transitions	1.00	5.00	3.61 ± 0.68	−0.60	1.30

Outcome variables/output levels	O1 Overall perceptions of patient safety	1.75	5.00	3.61 ± 0.54	0.17	0.11
	O2 Frequency of events reported	1.00	5.00	3.02 ± 1.02	0.28	−0.57

### 2.3. Pilot Study

A pilot survey was conducted via the convenience sampling approach, and 35 participants who met the inclusion and exclusion criteria were recruited. Survey completion times ranged from 251 to 1115 s. This step confirmed that the nurses comprehended the scale items and options and were able to complete the questionnaire accurately.

### 2.4. Data Collection

After approval was received from hospital authorities, the researchers distributed a link to an online electronic questionnaire to department heads and explained the inclusion and exclusion criteria for the survey. These department heads screened the qualified nurses in their units according to the inclusion and exclusion criteria, and then forwarded that link to the nurses. The nurses accessed and completed the questionnaires by clicking on the provided link. The questionnaire included standardized instructions, particularly on the first page; only nurses who met the criteria were asked to complete the questionnaire. The purpose and significance of this study and the completion requirements were also provided. The questionnaire stipulated that it would require at least 250 s to complete, and the instructions indicated that the questionnaire should be submitted within 1 week. After the questionnaires were collected, the first author anonymized the data by removing identifiable information, such as institution names, participants’ network names, and IP addresses. The qualifications of the nurses were subsequently checked and confirmed by reference demographic information, after which they were numbered. Approximately 1100 nurses from the four hospitals who met the inclusion criteria were included in this survey, and 823 responses were received, corresponding to a response rate ranging from 64.00% to 84.40% for the different hospitals. No nurses met the exclusion criteria and were excluded. After 31 questionnaires that did not meet the minimum completion time requirement were excluded, 792 valid responses remained, for an effective response rate of 96.23%.

### 2.5. Data Analysis

Data analysis was conducted with the assistance of SPSS 20.0 statistical software. The descriptive data are presented in terms of frequencies, percentages, means, and standard deviations.

The skewness coefficient (< 3) and kurtosis coefficient (< 8) were used to determine whether the variables followed a multivariate normal distribution [25]. The Pearson correlation coefficient, an independent sample *t* test, a one‐way analysis of variance, and nonparametric tests were used in this study. A multivariate linear regression analysis (enter method,*α*_in_ = 0.05, *α*_out_ = 0.10) was conducted by including significant factors and 12 dimensions included in the hospital survey pertaining to patient safety culture at a significance level of *p* < 0.05.

### 2.6. Ethical Considerations

This study received approval from the ethics committee of the first author’s institution (number 202503). This research was conducted via an online questionnaire, and an exemption from the need to obtain written informed consent was granted. Nevertheless, prior to the implementation of the survey, the participants were thoroughly briefed regarding the purpose, content, and significance of the study. They were also informed of the principle of voluntary participation in this research as well as their right to withdraw from this study at any time without prejudice. The participants provided implied consent by responding affirmatively to the following statement: “Having read the introduction to the purpose, content, and significance of this research, I agree to participate in this survey.”

## 3. Results

### 3.1. Demographic Characteristics of the Nurses

A total of 792 participants between the ages of 22 and approximately 59 years (the average age was 33.69 years; SD = 7.46) were ultimately included in the analysis. The results of the other descriptive analyses of the variables are presented in Table 2.

**TABLE 2:** Descriptive statistics and factors associated with three dimensions of the C‐BEDA (*n* = 792).

Variables	Categories	*n* (%)	Confidence and knowledge barriers (mean ± SD)	Institutional barriers (mean ± SD)	Psychological barriers (mean ± SD)	Financial concern barriers (mean ± SD)
Age (years)	≤ 25	85 (10.73)	2.68 ± 0.54	2.66 ± 0.48	3.20 ± 0.67	3.37 ± 0.76
	26∼30	246 (31.06)	2.68 ± 0.52	2.61 ± 0.56	3.08 ± 0.85	3.21 ± 0.92
	31∼35	194 (24.49)	2.66 ± 0.60	2.61 ± 0.63	3.08 ± 0.82	3.23 ± 0.89
	36∼40	121 (15.28)	2.49 ± 0.57	2.42 ± 0.62	3.24 ± 0.79	3.34 ± 0.83
	41∼45	69 (8.71)	2.48 ± 0.54	2.51 ± 0.61	3.12 ± 0.76	3.33 ± 0.86
	≥ 46	77 (9.73)	2.58 ± 0.56	2.45 ± 0.57	3.23 ± 0.88	3.35 ± 0.94

Statistics			3.318	3.052	1.074	0.872
*p*			0.006	0.010	0.373	0.500

Gender	Male	50 (6.31)	2.55 ± 0.70	2.56 ± 0.70	2.91 ± 0.86	3.03 ± 0.91
	Female	742 (93.69)	2.62 ± 0.55	2.56 ± 0.58	3.15 ± 0.81	3.29 ± 0.88

Statistics			0.766	0.056	1.996	2.047
*p*			0.447	0.955	0.046	0.020

Education	Secondary vocational school diploma	14 (1.77)	2.74 ± 0.50	2.66 ± 0.65	3.17 ± 1.14	3.46 ± 1.34
	College degree	235 (29.67)	2.65 ± 0.49	2.62 ± 0.50	3.06 ± 0.80	3.19 ± 0.88
	Bachelor’s degree	531 (67.04)	2.59 ± 0.57	2.51 ± 0.60	3.16 ± 0.80	3.30 ± 0.86
	Master’s degree or above	12 (1.52)	3.37 ± 0.71	3.33 ± 0.94	3.59 ± 0.97	3.62 ± 0.86

Statistics			8.534	15.788	2.136	1.664
*p*			< 0.001	0.001	0.094	0.173

Title	Junior	473 (59.72)	2.64 ± 0.50	2.60 ± 0.52	3.05 ± 0.78	3.20 ± 0.86
	Middle	233 (29.42)	2.61 ± 0.65	2.52 ± 0.68	3.24 ± 0.85	3.37 ± 0.89
	Senior	86 (10.86)	2.51 ± 0.60	2.45 ± 0.65	3.30 ± 0.86	3.42 ± 0.92

Statistics			2.052	11.246	14.760	4.129
*p*			0.129	0.004	0.001	0.016

Position—serve as a manager	Yes	260 (32.83)	2.54 ± 0.59	2.49 ± 0.62	3.11 ± 0.87	3.29 ± 0.95
	No	532 (67.17)	2.66 ± 0.54	2.59 ± 0.57	3.14 ± 0.78	3.27 ± 0.85

Statistics			−2.841	2.415	0.478	0.380
*p*			0.005	0.016	0.633	0.704

Length of the time employed (years)	≤ 5	229 (28.91)	2.70 ± 0.53	2.63 ± 0.54	3.14 ± 0.78	3.26 ± 0.83
	6∼10	230 (29.04)	2.65 ± 0.56	2.60 ± 0.60	3.00 ± 0.84	3.18 ± 0.92
	11∼15	154 (19.44)	2.61 ± 0.59	2.55 ± 0.62	3.20 ± 0.78	3.34 ± 0.88
	16–20	76 (9.60)	2.37 ± 0.50	2.38 ± 0.63	3.31 ± 0.78	3.38 ± 0.89
	≥ 21	103 (13.01)	2.56 ± 0.57	2.45 ± 0.58	3.18 ± 0.86	3.35 ± 0.89

Statistics			5.837	3.839	2.703	1.302
*p*			< 0.001	0.004	0.030	0.268

Marital status	Married	633 (79.92)	2.60 ± 0.56	2.52 ± 0.60	3.12 ± 0.82	3.26 ± 0.88
	Unmarried	144 (18.18)	2.72 ± 0.55	2.71 ± 0.51	3.19 ± 0.76	3.34 ± 0.86
	Other (divorced/widowed)	15 (1.90)	2.37 ± 0.53	2.65 ± 0.44	3.07 ± 1.16	3.13 ± 1.11

Statistics			3.953	15.843	0.904	0.625
*p*			0.020	< 0.001	0.636	0.536

Form of labour contract	Civil servant	532 (67.17)	2.62 ± 0.51	2.58 ± 0.55	3.07 ± 0.79	3.22 ± 0.87
	other	260 (32.83)	2.61 ± 0.64	2.52 ± 0.66	3.27 ± 0.85	3.39 ± 0.89

Statistics			−0.266	1.183	3.260	2.507
*p*			0.790	0.237	0.001	0.012

Hospital level	Secondary level	365 (46.09)	2.57 ± 0.53	2.53 ± 0.55	3.06 ± 0.80	3.21 ± 0.86
	Third level	427 (53.91)	2.66 ± 0.58	2.58 ± 0.62	3.20 ± 0.82	3.33 ± 0.90

Statistics			−2.282	−1.182	−2.313	−2.020
*p*			0.023	0.238	0.021	0.044

Working department	Internal medicine	216 (27.27)	2.71 ± 0.54	2.65 ± 0.59	3.16 ± 0.80	3.31 ± 0.86
	Surgery	145 (18.31)	2.52 ± 0.57	2.52 ± 0.63	3.21 ± 0.77	3.40 ± 0.84
	Outpatient	65 (8.21)	2.80 ± 0.48	2.67 ± 0.57	3.24 ± 0.65	3.35 ± 0.75
	Emergency	44 (5.55)	2.54 ± 0.58	2.56 ± 0.42	3.05 ± 0.88	3.25 ± 0.93
	Gynaecology/paediatrics	84 (10.61)	2.71 ± 0.53	2.56 ± 0.61	3.08 ± 0.88	3.20 ± 0.98
	Intensive care unit	25 (3.16)	2.61 ± 0.51	2.44 ± 0.54	2.93 ± 0.80	3.06 ± 0.65
	Other departments	213 (26.89)	2.52 ± 0.57	2.48 ± 0.59	3.08 ± 0.85	3.20 ± 0.93

Statistics			4.539	2.208	0.981	1.227
*p*			< 0.001	0.040	0.437	0.290

Adverse event report times	None	28 (3.53)	3.29 ± 0.53	2.92 ± 0.95	3.27 ± 1.10	3.37 ± 0.99
	1–2 times	689 (86.99)	2.60 ± 0.54	2.54 ± 0.55	3.13 ± 0.79	3.26 ± 0.87
	3–5 times	45 (5.68)	2.53 ± 0.57	2.56 ± 0.68	3.04 ± 0.91	3.23 ± 0.97
	6–10 times	17 (2.15)	2.51 ± 0.63	2.53 ± 0.77	3.16 ± 0.71	3.38 ± 0.67
	≥ 11 times	13 (1.65)	2.68 ± 0.71	2.61 ± 0.67	3.36 ± 0.94	3.73 ± 0.86

Statistics			11.159	10.543	0.597	1.073
*p*			< 0.001	0.032	0.665	0.369

*Note:* In China, professional titles in the nursing field are classified into three levels: junior (nurse, senior nurse), middle (nurse in charge), and senior (associate chief nurse, chief nurse).

### 3.2. Total Mean Score for Each Dimension of the C‐BEDA Tool

The scores pertaining to the various dimensions of the C‐BEDA tool are presented in Table 1.

### 3.3. Total Mean Score for Each Dimension of the HSOPSC

The scores pertaining to the various dimensions of the HSOPSC are presented in Table 1.

### 3.4. Correlation Between the C‐BEDA Tool and the HSOPSC

The correlations between patient safety culture dimensions and medical error disclosure barriers are presented in Table 3.

**TABLE 3:** Correlations between patient safety culture dimensions and medical error disclosure barriers.

Dimensions	Confidence and knowledge barriers	Psychological barriers	Institutional barriers	Financial concern barriers
U1 Supervisor/manager expectations & actions promoting patient safety	−0.360^∗∗^	−0.082^∗^	−0.394^∗∗^	−0.077^∗^
U2 Organizational learning‐ continuous improvement	−0.224^∗∗^	−0.002	−0.237^∗∗^	0.025
U3 Teamwork within units	−0.226^∗∗^	−0.073^∗^	−0.266^∗∗^	−0.053
U4 Communication openness	−0.260^∗∗^	−0.094^∗∗^	−0.234^∗∗^	−0.088^∗^
U5 Feedback & communication about error	−0.282^∗∗^	0.070^∗^	−0.251^∗∗^	0.052
U6 Nonpunitive response to errors	−0.305^∗∗^	−0.223^∗∗^	−0.268^∗∗^	−0.223^∗∗^
U7 Staffing	−0.147^∗∗^	−0.152^∗∗^	−0.222^∗∗^	−0.148^∗∗^
H1 Management support for patient safety	−0.391^∗∗^	−0.136^∗∗^	−0.477^∗∗^	−0.123^∗∗^
H2 Teamwork across units	−0.415^∗∗^	−0.182^∗∗^	−0.417^∗∗^	−0.136^∗∗^
H3 Hand‐offs & transitions	−0.374^∗∗^	−0.182^∗∗^	−0.352^∗∗^	−0.137^∗∗^
O1 Overall perceptions of patient safety	−0.312^∗∗^	−0.121^∗∗^	−0.298^∗∗^	−0.115^∗∗^
O2 Frequency of events reported	−0.122^∗∗^	0.067	−0.096^∗∗^	0.049

^∗^ indicates *p* < 0.05.

^∗∗^ indicates *p* < 0.001.

### 3.5. Results of the Single‐Factor Analysis

Age, sex, and other factors were included as independent variables. Barriers pertaining to confidence and knowledge barriers, institutional barriers, psychological barriers, and financial concern barriers were included as dependent variables, and a single‐factor analysis was conducted. The results of this analysis are presented in Table 2.

### 3.6. Results of the Multiple Linear Regression Analysis

The demographic and work‐related characteristics associated with these dependent variables were assigned first. Categorical variables were quantified as follows: binary variables were encoded using 0 and 1, ordinal variables were encoded as continuous variables, and multiclass categorical variables were represented through dummy variable encoding to fully capture all category distinctions. For these allocation details, please see Table 4. These assigned variables were subsequently included as control variables. The various dimensions at the unit level and the hospital level as well as outcome variables pertaining to patient safety culture were gradually incorporated as independent variables into the regression equation; please see Table 5. The results of the multivariate linear regression analysis are presented in Table 6.

**TABLE 4:** The assignment of independent variables included in the multiple linear regression analysis.

Variables	Assignment
Age (years)	≤ 25 = 1, 26∼30 = 2, 31∼35 = 3, 36∼40 = 4, 41∼45 = 5, ≥ 46 = 6
Gender	Female = 1, male = 0
Education	Secondary vocational school diploma = 1, college degree = 2, bachelor’s degree = 3, master’s degree or above = 4
Title	Junior = 1, middle = 2, senior = 3
Serve as a manager	Yes = 1, no = 0
Length of the time employed	≤ 5 years = 1, 6∼10 years = 2, 11∼15 years = 3, 16–20 years = 4, ≥ 21 years = 5
Marital status	Married = 1, other = 0
Form of labour contract	Civil servant = 1, other = 0
Hospital level	Secondary level = 1, third level = 2
Working department	Set dummy variables with internal medicine as the reference, X1 = surgery (0, 1); X2 = outpatient (0, 1); X3 = emergency (0, 1); X4 = gynaecology/paediatrics (0, 1); X5 = intensive care unit (0, 1); X6 = other departments (0, 1)
Adverse event report times	None = 0, 1–2 times = 1, 3–5 times = 2, 6–10 times = 3, ≥ 11 times = 4

**TABLE 5:** The sequence, steps, and results of multiple linear regression analysis.

Model	Dependent variable	The sequence and steps of including independent variables	*R*	*R* ^2^	Adjusted *R* ^2^	*F*	*p*	Durbin–Watson
1	Confidence and knowledge barriers	Step1 age, education, serve as a manager, length of the time employed, marital status, hospital level, working department, adverse event report times	0.227	0.051	0.041	4.716	< 0.001	1.831
2	Confidence and knowledge barriers	Step2 step1 and U1∼U7	0.467	0.218	0.202	13.540	< 0.001	1.804
3	Confidence and knowledge barriers	Step3 step2 and H1∼H3	0.521	0.272	0.254	15.148	< 0.001	1.827
4	Confidence and knowledge barriers	Step4 step3 and O1, O2	0.526	0.277	0.257	14.060	< 0.001	1.819
5	Institutional barriers	Step1 age, title, serve as a manager, length of the time employed, marital status, working department, adverse event report times	0.201	0.041	0.026	2.743	0.001	1.870
6	Institutional barriers	Step2 step1 and U1∼U7	0.464	0.215	0.196	11.136	< 0.001	1.845
7	Institutional barriers	Step3 step2 and H1∼H3	0.534	0.285	0.265	13.941	< 0.001	1.878
8	Institutional barriers	Step4 step3 and O1, O2	0.536	0.287	0.265	12.871	< 0.001	1.870
9	Psychological barriers	Step1 gender, title, length of the time employed, form of labor contract, hospital level	0.181	0.033	0.027	5.344	< 0.001	1.915
10	Psychological barriers	Step2 step1 and U1, U3∼U7	0.319	0.102	0.089	8.049	< 0.001	1.935
11	Psychological barriers	Step3 step2 and H1∼H3	0.339	0.115	0.099	7.219	< 0.001	1.940
12	Psychological barriers	Step4 step3 and O1	0.340	0.115	0.098	6.729	< 0.001	1.940
13	Financial concern barriers	Step1 gender, title, form of labour contract, hospital level	0.123	0.015	0.013	6.028	0.003	1.864
14	Financial concern barriers	Step2 step1 and U1, U4, U6, U7	0.255	0.065	0.061	18.222	< 0.001	1.892
15	Financial concern barriers	Step3 step2 and H1∼H3	0.255	0.065	0.061	18.222	< 0.001	1.892
16	Financial concern barriers	Step4 step3 and O1	0.257	0.066	0.061	13.887	< 0.001	1.885

**TABLE 6:** The results of medical error disclosure barriers’ multiple linear regression analysis.

Models and variables	*B*	SE	*β*	*t*	*p*	VIF
Model 3 of confidence and knowledge barriers						
Constant	4.675	0.134	—	34.994	< 0.001	—
Surgery	−0.147	0.047	−0.102	−3.132	0.002	1.098
Other departments	−0.146	0.041	−0.116	−3.576	< 0.001	1.097
U6	−0.096	0.029	−0.120	−3.369	< 0.001	1.310
U5	−0.084	0.026	−0.116	−3.205	0.001	1.359
U4	−0.065	0.029	−0.080	−2.214	0.027	1.354
H2	−0.204	0.041	−0.217	−4.993	< 0.001	1.971
H3	−0.102	0.036	−0.123	−2.809	0.005	2.001
Model 8 of institutional barriers						
Constant	4.974	0.215	—	23.161	< 0.001	—
Age	−0.066	0.039	−0.076	−1.681	0.093	2.189
Title	−0.019	0.020	−0.033	−0.943	0.346	1.333
Serve as a manager	−0.037	0.042	−0.030	−0.873	0.383	1.229
Length of the time employed	−0.048	0.043	−0.045	−1.125	0.261	1.686
Marital status	0.103	0.043	0.080	2.406	0.016	1.184
U2	0.006	0.055	0.005	0.115	0.909	1.904
Model 10 of psychological barriers						
Constant	3.491	0.299	—	11.662	< 0.001	—
Gender	−0.064	0.060	−0.041	−1.062	0.289	1.309
Title	−0.007	0.056	−0.006	−0.135	0.893	1.492
Length of the time employed	−0.253	0.049	−0.216	−5.147	< 0.001	1.533
U1	0.026	0.063	0.018	0.405	0.685	1.708
U4	−0.147	0.048	−0.124	−3.051	0.002	1.430
Model 14 of financial concern barriers						
Constant	3.741	0.196	—	19.036	< 0.001	—
Title	0.126	0.044	0.098	2.842	0.005	1.000
Gender	0.274	0.125	0.076	2.194	0.029	1.000
U6	−0.283	0.044	−0.223	−6.479	< 0.001	1.000

*Note:* Variance inflation factor, VIF. Model 3: *R* = 0.521, *R* ^2^ = 0.272, adjusted *R* ^2^ = 0.254, *F* = 15.148, *p* < 0.001, Durbin–Watson = 1.827. Model 8: *R* = 0.536, *R* ^2^ = 0.287, adjusted *R* ^2^ = 0.265, *F* = 12.871, *p* < 0.001, Durbin–Watson = 1.870. Model 10: *R* = 0.319, *R* ^2^ = 0.102, adjusted *R* ^2^ = 0.089, *F* = 8.049, *p* < 0.001, Durbin–Watson = 1.935. Model 10: *R* = 0.255, *R* ^2^ = 0.065, adjusted *R* ^2^ = 0.061, *F* = 18.222, *p* < 0.001, Durbin–Watson = 1.892.

### 3.7. Results Regarding Other Variables

The other variables included in the C‐BEDA tool are associated with six items, and the results pertaining to each item are presented in Table 7.

**TABLE 7:** The research results of other variables.

Item	Strongly disagree	Disagree	Neutral	Agree	Strongly agree
13. I am uncertain about how to report errors/mistakes.	84 (10.61)	397 (50.13)	191 (24.12)	103 (13.00)	17 (2.14)
14. My institution provides peer support services that help providers deal with the emotional consequences of error.	19 (2.40)	110 (13.90)	298 (37.62)	314 (39.64)	51 (6.44)
16. I would like to be included in the error disclosure process in the event I am involved in a medical error.	3 (0.38)	31 (3.91)	203 (25.63)	482 (60.86)	73 (9.22)
17. The physician is the ultimate one responsible for disclosing the medical error, regardless of his/her involvement in the error.	13 (1.64)	102 (12.88)	249 (31.44)	366 (46.21)	62 (7.83)
18. I am afraid of being blamed for a medical error if I am not present during the disclosure conversation with a patient and/or patient’s family.	26 (3.28)	197 (24.87)	265 (33.46)	272 (34.34)	32 (4.05)
19. Other non‐physician healthcare providers (e.g. nurses, pharmacists, etc.) should be included in the disclosure conversation if they contributed to the occurrence of the error.	8 (1.01)	75 (9.47)	230 (29.04)	413 (52.15)	66 (8.33)

## 4. Discussion

This study investigated 792 nurses in China by using the C‐BEDA tool and the HSOPSC to explore the relationship between barriers to medical error disclosure and hospitals’ patient safety culture. The results revealed that financial concerns emerged as the predominant barrier to medical error disclosure, followed closely by psychological barriers, and then deficits in confidence and knowledge. Barriers to disclosing medical errors are indeed related to nurses’ perceptions of their hospitals’ patient safety culture. These findings underscore that fostering a robust patient safety culture enhances nurses’ confidence and capacity for error disclosure. Specifically, the barrier of nurses’ financial concern was negatively associated with the perception of nonpunitive responses to errors. Psychological barriers were negatively related to supervisor expectations, actions promoting patient safety, and communication openness. Accordingly, when the levels of transparency and openness in communication are higher, this situation can reduce psychological barriers and make it easier for nurses to disclose errors. Leaders in hospitals should prioritize the establishment of a patient safety culture at the unit level, particularly with respect to nonpunitive management and openness in communication. In total, to encourage nurses to disclose medical errors effectively, it is imperative first to address their financial concerns and second to provide them with comprehensive disclosure training alongside psychological support.

Studies have revealed that barriers to disclosing medical errors are hindered by fears of litigation and legal liability, apprehension about job loss, a lack of apology protection laws, inadequate institutional backing, and the absence of standardized error disclosure guidelines [16, 26–29]. However, in previous studies, psychological barriers stand as the foremost obstacles preventing nurses from disclosing errors [30]. This study revealed that the financial concerns experienced by nurses ranked highest, which may be due to the underdeveloped medical compensation and insurance systems that characterize the southwestern region of China. In China, the medical liability insurance system remains in an exploratory phase; institutional coverage represents the predominant model, and no practice by which individual nurses can purchase insurance has been established [31, 32]. Although specific models vary across regions [33], nurses are invariably involved when medical errors result in economic compensation. Hospitals typically assess the nurse’s level of responsibility and, according to their internal management policies, require the nurse to bear a certain proportion of the compensation amount, as well as an additional fine. These are regarded as the manifestations of punishment. Given that these financial burdens are not covered by any form of insurance, economic consequences represent a significant barrier for nurses in the context of disclosing medical errors.

Consistent with the findings of this study, psychological disorders pose a substantial challenge for nurses [30]. The data in Table 6 reveal that gender, title, tenure, supervisor expectations, actions taken to promote patient safety, and communication openness collectively explain 10.2% of the variance in the psychological barriers faced by nurses. These findings demonstrate a significant association between patient safety culture and psychological disorders among nurses. Managers should genuinely consider staff suggestions for improving patient safety, actively praise staff for adhering to patient safety procedures, and never overlook patient safety concerns. Moreover, managers should actively encourage and support nurses in facing mistakes candidly, demonstrating sound professional judgment. Managerial support and affirmation can not only prevent misunderstandings regarding management intentions but also cultivate positive attitudes towards error disclosure. In addition, a firm commitment to nonpunitive management practices, an equitable perspective on errors, and deep respect for nursing professionals may significantly alleviate the concerns and anxiety associated with error reporting [34].

Increasing individuals’ confidence in disclosing errors has been identified as the most difficult task [35]. This study revealed that both unit‐level and hospital‐level patient safety culture improved confidence and knowledge among nurses. Although the obligation to disclose errors has become the target of industry consensus, various differences in the details remain [22]. Through several key improvements, such a consensus can be gradually reached in hospital clinical practice. First, hospital management should provide a work climate that promotes patient safety and indicates that patient safety is a top priority. Second, error management can be enhanced through communication openness, feedback about errors, and nonpunitive responses to errors. In addition, cooperation and coordination should be promoted across units. A relevant study reported more tips, including sharing errors openly with the goal of providing learning opportunities and transforming individuals’ perspectives on the process of overcoming and learning from errors [36]. These approaches can help nurses feel better about changes in hospital management and prevent them from misunderstanding the organization’s management intentions in the context of error event reports. A good atmosphere for organizational learning and improvement cannot be established without the active participation of all unit members. Additionally, error disclosure education and training and continuous positive improvement actions are essential.

The dimensions of patient safety culture are negatively correlated with barriers pertaining to confidence, psychological barriers, and institutional barriers, in which context, the strongest correlation is observed with institutional barriers. These findings suggest that nurses’ perceived disclosure barriers are closely linked to hospital organizational management. Age, title, managerial position, tenure, marital status, and continuous improvement in organizational learning explained 28.7% of the variance in nurses’ institutional barriers. In this context, unmarried nurses perceive higher levels of institutional barriers, which require attention from hospital managers. This finding may be related to the fact that unmarried nurses are generally younger, have less social experience and work experience, and lack diverse forms of family support. Studies have reported that various organizational factors, such as inefficient reporting systems and unequal treatment of individuals who report errors, can prevent nurses from disclosing errors [21]. Young healthcare workers tend to adopt negative attitudes towards the notion of a safety climate and their own responsibility for error disclosure, which may be attributed to the concealment of medical errors from patients, an imperfect feedback communication mechanism, and inadequate organizational learning and improvement [17]. By progressively incorporating the various dimensions of patient safety culture at the unit, hospital, and output levels, we revealed that unit‐level safety culture has the most significant effect on confidence‐related barriers, psychological barriers, and institutional barriers. This insight can facilitate a re‐evaluation of the role played by the patient safety culture in efforts to promote error disclosure and justify increased investment in the development of a unit safety culture, thereby potentially enhancing the effectiveness of disclosure practices.

In this study, 96.5% of the nurses reported errors, and more than 60% of the nurses were aware of how to report mistakes. These findings might be related to the fact that the hospital attaches great importance to training in error reports. However, 69% of the nurses desired to be present during the disclosure conversation because they were afraid of being blamed for medical errors. This finding may be due to their need to defend their position [37]. Only 4% of the nurses wanted to be included in the error disclosure process. Obviously, nurses face complex obstacles pertaining to disclosure. Both the promotion of disclosure policies and training in the process of disclosure remain inadequate [38].

## 5. Limitations of This Study

This research employed convenience sampling to select four hospitals in the southwestern region of China. This sampling approach could introduce selection bias, and the geographic concentration may constrain the generalizability of the findings. Future studies should adopt stratified random sampling techniques and incorporate hospitals from diverse regions or countries to bolster external validity. Additionally, the response rate among nurses at one hospital was only 64%, which is relatively low. Consequently, the data from this hospital might inadequately represent the experiences of the entire group of nurses. Kurtosis and skewness were used to determine whether the research variables followed a normal distribution; however, this approach represents a less stringent criterion with respect to multivariate normality. Additionally, the sample included a limited number of nurses who were technical school graduates or who had obtained master’s degrees and above; however, these subgroups were analyzed separately. Furthermore, certain dimensions of patient safety culture that are not directly related to disclosure barriers were excluded from the multiple linear regression analysis, although this exclusion does not imply that these dimensions do not influence the barriers to disclosure.

## 6. Conclusions

The primary obstacles faced by nurses include financial concerns before the disclosure of errors, followed by psychological barriers. To encourage nurses to disclose medical errors effectively, hospitals must first address nurses’ concerns regarding economic issues and provide them with comprehensive training in error disclosure alongside psychological support. Additionally, hospitals should prioritize the establishment of a patient safety culture at the unit level, particularly with respect to organizational learning, continuous improvement, and openness in communication.

## 7. Implications for Nursing Management

Nurses who disclose medical errors to patients need economic and psychological support.

Hospitals and nursing associations should facilitate access to professional liability insurance for all nurses.

Hospitals should provide training in the disclosure of medical errors, including with respect to relevant policies, procedures, standards, and communication skills.

Superior managers should increase their investment in the construction of patient safety culture at the unit level as well as the support they provide in this context, particularly concerning the dimensions of communication openness, nonpunitive responses to errors, and management support for patient safety.

During the process of implementing disclosure, superior managers should focus on the task of establishing an atmosphere that emphasizes organizational learning, continuous improvement, and openness in communication.

During the process of implementing disclosure, special attention should be given to the psychological state of unmarried and junior nurses.

## Author Contributions

Conceptualization and study design, data collection, and project administration: Gui‐Ru Chen and Rong‐Rong Huang.

Analysis and interpretation: Gui‐Ru Chen and Xin Luo.

Manuscript drafting and critical revision: Gui‐Ru Chen, Xin Luo, and Rong‐Rong Huang.

Manuscript critical revision and literature review: Heng‐Yu Xiong, Xiao‐Min Ding, and Shi‐Hua Pan.

All authors have contributed significantly to the work and agree to be held accountable for its content.

## Funding

Funding for this study was provided by the institutions/organizations of the first and corresponding authors. These institutions and organizations are nonprofit and do not interfere with the process and results of research. This research was supported by the Science and Technology Department of Sichuan Province (grant number 2020JDR0368), the Nursing Research Fund of Guizhou Medical University Affiliated Hospital (grant number GYHLB202202), the People’s Hospital of Aba Tibetan and Qiang Autonomous Prefecture Program (grant numbers 202214 and 202314), the Health Commission of Sichuan Province Medical Science and Technology Program (grant number 24QNMP044), and the Nursing Research Project of Sichuan Provincial Nursing Association (grant number H24011).

## Conflicts of Interest

The authors declare no conflicts of interest.

## Data Availability

The anonymized data that support the findings of this study are available from the Science Data Bank (DOI 10.57760/sciencedb.30294, CSTR 31253.11.sciencedb.30294).
